# JGMS EDITOR’S CHOICE ARTICLES OF 2022: BIOLOGIC AND SOCIAL INFLUENCES ON COGNITIVE AND PHYSICAL AGING

**DOI:** 10.1093/geroni/igad104.0713

**Published:** 2023-12-21

**Authors:** Lewis Lipsitz, Peggy Cawthon

## Abstract

This symposium will present four 2022 “Editor’s Choice” articles from the Journal of Gerontology Medical Sciences that focus on the biological and social influences on cognitive and physical aging. Mercedes Sotos-Prieto and her colleagues, in their article “A Mediterranean Lifestyle and Frailty Incidence in Older Adults: The Seniors-ENRICA-1 Cohort” examine the association between a Mediterranean lifestyle, which included diet, customs, and traditions, and frailty incidence in older adults. Mark Espeland and his colleagues, in “Racial/Ethnic Disparities in Alzheimer’s Disease Risk: Role of Exposure to Ambient Fine Particles” used unique data from the Women’s Health Initiative Memory Study to investigate this previously unstudied relationship between racial disparities, ambient fine particle exposure, and risk of Alzheimer’s. “Trans-Ethnic Meta-Analysis of Interactions Between Genetics and Early-Life Socioeconomic Context on Memory Performance and Decline in Older Americans,” written by Jessica Faul and colleagues, examines whether gene regions previously associated with cognitive performance, dementia, and related traits had an interaction with childhood socioeconomic context on memory performance or decline. Lingxiao He and coauthors, in their article, “Prospective Associations of Plasma Growth Differentiation Factor 15 With Physical Performance and Cognitive Functions in Older Adults” examines the relationship between Plasma GDF15, lower-limb physical performance, and global cognitive function in older adults. Our discussant, Peggy Cawthon, will highlight commonalities and lessons learned from these studies.

